# Land surface temperature evolution in rapidly urbanizing areas of Southeast Asia: Studies from Vietnam and Cambodia

**DOI:** 10.1371/journal.pone.0328750

**Published:** 2025-10-15

**Authors:** Bijeesh Kozhikkodan Veettil, Juliana Costi, Vanna Teck, Vikram Puri

**Affiliations:** 1 Laboratory of Ecology and Environmental Management, Science and Technology Advanced Institute, Van Lang University, Ho Chi Minh City, Vietnam; 2 Faculty of Applied Technology, Van Lang School of Technology, Van Lang University, Ho Chi Minh City, Vietnam; 3 Instituto de Oceanografia, Universiade Federal do Rio Grande, Rio Grande, Brazil; 4 Faculty of Science and Technology, Svay Rieng University, Svay Rieng City, Cambodia; 5 School of Computer Science, Duy Tan University, Da Nang, Vietnam; 6 Institute of Research and Development, Duy Tan University, Da Nang, Vietnam; Hanoi University of Mining and Geology, VIET NAM

## Abstract

Land surface temperature (LST) is one of the crucial variables in urban microclimate studies. Satellite-based thermal data and vegetation indices, like the normalized difference vegetation index (NDVI), help to understand changes in LST and the development of urban heat islands (UHI). We analyzed the variations in LST and vegetation coverage in two rapidly urbanizing provinces, located in southern Vietnam and Cambodia, respectively, over the 10 years from 2013 to 2025. Additionally, complementary ERA5 Interim air temperature data were also utilized. The satellite and *in situ* data analysis have been used to understand the impacts of urbanization on LSTs. Spatiotemporal changes in NDVI showed rapid urbanization in the eastern region of Battambang city (39.2 km^2^ to 47.8 km^2^) and throughout the southern areas of Binh Duong Province (387 km^2^ to 464.3 km^2^). Time-series analysis indicated a consistent increase in LST in both study sites. There has been a notable increase in minimum LST since 2017 in the entire city of Battambang, whereas the central area of Battambang has become consistently warmer after 2020. The minimum estimated LST in Battambang varied between 16.1 °C and 28.58 °C (and increased 0.35 °C per year), whereas the maximum LST varied between 29.2 °C to 40.23 °C (and increased 0.36 °C per year). The LST in southern Binh Duong increased gradually during the study period, primarily due to rapid urbanization and vegetation loss. The minimum estimated LST in Binh Duong varied between 13.2 °C to 24.73 °C (and increased 0.26 °C per year), whereas the maximum LST varied between 34.6 °C to 41.3 °C (and increased 0.024 °C per year). The outcome of this study holds considerable importance, as the phenomenon of UHI formation has been documented in rapidly expanding cities and impervious surfaces globally, especially in Southeast Asia.

## 1. Introduction

The physics of land surface processes is highly influenced by land surface temperatures (LST) [1]. LST is a crucial parameter in the study of urbanization and climate change [2]. Several studies have analyzed the connection between land use changes (rapid urbanization in particular) and LST variations [3,4]. Urban structures with higher heat storage capacity and decreased evaporation in cities lead to higher minimum land surface temperatures (LST) [5]. Urbanization’s impact on LST attracted the scientific community, as it is believed to influence the local climate in cities compared to their neighborhoods [6]. These variations in microclimate between urban areas and their surroundings have been studied based on air temperature [7] or LST [8] measurements. LST data provides a reliable measure for assessing urban microclimate patterns [9]. Satellite-based thermal infrared imagery has been used for LST retrieval by applying radiative transfer equations since the 1970s [1].

Global urbanization has substantially impacted environmental and climate systems, intensifying climate change effects [10]. For this reason, urbanization has attracted environmental scientists in the 21^st^ century [4]. Urbanization is a key anthropogenic activity that negatively impacts ecosystems, biodiversity, and urban microclimate [11,12]. Rapid urban growth that lacks adequate planning is anticipated to aggravate these harmful consequences [13]. Therefore, a recent study by Maheshwari et al. [14] suggested considering urbanization as one of the parameters in developing climate change adaptation strategies and policies to deal with future climate change scenarios.

The UHI effect is characterized by increased air and ground temperatures in cities as compared to adjacent rural areas [15], has been studied by scientists worldwide in the past few decades. Large cities and areas undergoing rapid urbanization may experience higher temperatures compared to rural regions because of the formation of UHI [16]. The magnitude and geographic concentration of UHI-associated risks can be evaluated using UHI intensity and spatial distribution data [17]. Urban land cover types significantly affect UHI formation. Land cover modifications can be evaluated through the association between LST and vegetation indices like the Normalized Difference Vegetation Index (NDVI) [18]. Therefore, the formation and development of UHI can be analyzed by measuring LST measurements using satellite or unmanned aerial vehicle (UAV) imagery with thermal infrared information, which can be used as input for eco-friendly urban planning and development initiatives [4,19].

Time-series LST values from remote sensing are considered as a proxy measurement in understanding the formation of UHIs [4,12,20–23]. Satellite data with thermal infrared wavelengths, such as the Landsat series (except Multispectral Scanner-MSS) and ASTER, have proven their capability in measuring LST from space [4,12,23]. Different computational approaches, including automatic, semi-automatic, and machine learning algorithms, have been developed for determining LST and UHI intensity. In addition to rapid measurements, satellite data offer cost-effective solutions for measuring LST compared to field measurements, as logistic expenses are high in the latter [4,23], particularly when time-series analysis is required. The availability of thermal infrared in the formation of satellite data has revolutionized UHI research [4].

Southeast Asia has been experiencing one of the fastest rates of urbanization in the world, with Vietnam and Cambodia at the forefront of this transformation. Rapid population growth, industrialization, and infrastructure expansion have driven large-scale land use and land cover (LULC) changes in major cities such as Hanoi, Ho Chi Minh City, Phnom Penh, and Siem Reap. These changes are accompanied by the replacement of natural landscapes with impervious surfaces, which have significant implications for the surface energy balance, urban climate, and thermal environment [24,25]. One of the most visible consequences is the intensification of LST, which is closely linked to the UHI effect [5]. The study of LST evolution in such rapidly urbanizing contexts is crucial for several reasons. First, elevated surface and air temperatures contribute to increased energy demand, particularly for cooling, placing additional stress on already strained urban infrastructures [25,26]. Second, the intensification of LST has public health implications, particularly in tropical Southeast Asia, where heat stress and vulnerability to extreme weather events are already high [27]. Third, in the context of climate change, urban areas act as hotspots that exacerbate warming trends, thereby creating a dual challenge of global and local drivers of temperature rise [28]. Understanding the evolution of LST in Vietnam and Cambodia is therefore vital for developing sustainable urban planning and climate adaptation strategies. The spatiotemporal variations in land surface temperatures in two rapidly urbanizing provinces, each from Vietnam and Cambodia, were analyzed during the period between 2013 and 2025 using satellite data having thermal infrared imagery and ERA5 air temperature data. This analysis offers a comprehensive insight into the effect of alterations in land use and land cover (LULC) on the formation of UHIs and the potential consequences of other human-induced processes on the development of UHIs.

From a scientific perspective, the motivation for this study arises from both data availability and methodological innovation. Landsat imagery, with its medium spatial resolution, has been widely used for historical LST retrieval and offers valuable insights into spatial variations of temperature across heterogeneous urban landscapes [29]. On the other hand, ERA5, the state-of-the-art atmospheric reanalysis dataset, provides long-term, temporally consistent records of air temperature at a global scale [30]. By combining Landsat’s spatial resolution with ERA5’s temporal resolution, this study seeks to overcome the individual limitations of each dataset, offering a more comprehensive understanding of LST dynamics in Southeast Asia. Vietnam and Cambodia provide particularly compelling case studies due to their contrasting stages of urban development and regional climatic diversity. Vietnam, with its major metropolitan centers, has witnessed extensive urban sprawl and rapid land conversion. Cambodia, while urbanizing at a slightly slower pace, faces challenges of unregulated growth, infrastructure constraints, and climate vulnerability. Examining both contexts together allows for comparative insights into how different urbanization trajectories shape thermal environments. In addition, the rationale for this study is rooted in the policy relevance of LST research. Despite the mounting evidence of heat-related challenges, urban climate considerations are often underrepresented in planning policies in Southeast Asia. By providing robust, spatially explicit evidence of LST trends, this study can inform decision-makers, urban planners, and climate adaptation programs in Vietnam, Cambodia, and the wider ASEAN region. It contributes to filling a critical knowledge gap at the intersection of urbanization, climate science, and sustainable development. This study is motivated by the urgent need to understand how rapid urbanization in Southeast Asia influences LST, the potential of integrating Landsat and ERA5 datasets to provide complementary insights, and the pressing policy demand for climate-smart urban planning. By focusing on Vietnam and Cambodia, the research highlights both the scientific and societal importance of addressing urban thermal environments in a region undergoing profound transformation.

## 2. Study area

**Fig 1:**
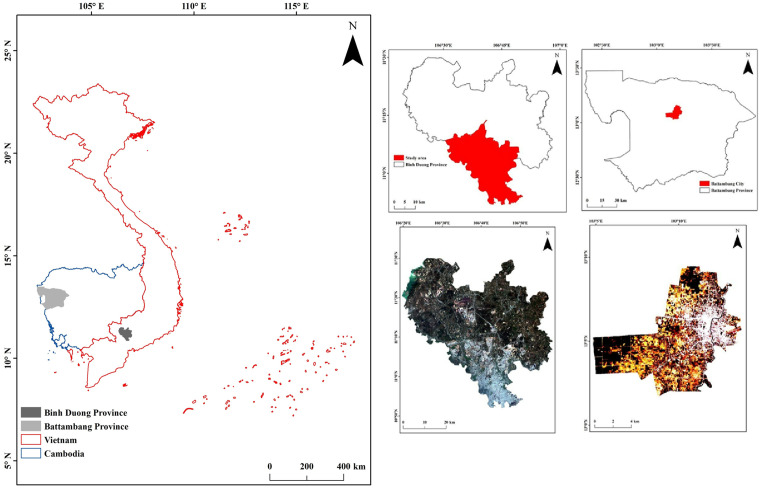
Study sites considered in Vietnam and Cambodia. USGS EROS (Earth Resources Observatory and Science (EROS) Center) (public domain): http://eros.usgs.gov/#.

It is well known that industrialization and urbanization have reduced poverty in rural areas of Vietnam [31]. Such areas that underwent rapid urbanization were previously dependent on agriculture for income, and later (since 2010) these land areas were acquired by the government for urbanization for the socioeconomic transition in Vietnam [32]. The present study considered two rapidly urbanizing provinces from Vietnam and Cambodia, Binh Duong and Battambang, respectively (Fig 1). Several recent studies discussed highly urbanized areas in major cities such as Hanoi (Vietnam) and Phnom Penh (Cambodia) [33,34]. However, not many studies have considered small-scale areas undergoing rapid urbanization in recent decades. This knowledge gap is covered in the present study. The quality of environmental elements such as soil, water, and air has worsened in large urban and semi-urban areas over the past few decades because of rapid urbanization [4,23].

Several studies conducted in Vietnam, including those in Hanoi and Ho Chi Minh City, observed the formation and development of the UHI effect [16,21,23,35] (Hoan et al. 2018; Son et al. 2017; Van and Bao 2020; Veettil and Van 2023), and small urbanized areas, such as Danang and Hue [4]. Son et al. [21] reported that the LST values in Ho Chi Minh City changed from 31 °C in the 1990s to 32^o^C in the 2010s. Veettil and Van [23] found a modest diminution in LST values in Ho Chi Minh City during the prolonged lockdown measures implemented because of COVID-19.

In the recent study, LST patterns in two rapidly urbanizing provinces, each from Vietnam and Cambodia, were analyzed during the period between 2013–2025. For this purpose, two urban areas were considered for estimating LST variations during the study period from satellite imagery. In addition to LST variations, this study also considered the vegetation dynamics in the study areas during the same period by using vegetation indices.

### 2.1. Binh Duong, Vietnam

Binh Duong Province, spanning an area of 2,694 square kilometers, is situated in southeastern Vietnam, renowned for its industrial significance. Thu Dau Mot City (10°58′N, 106°39′E) is the capital of Binh Duong Province in southeastern Vietnam. The advancement of industries has significantly added to the province’s economic growth. In parallel with the expansion of the urbanized areas and population growth, the land cover and land use in this region also changed significantly over the past three decades. It is worth noting that 77% of the land cover is used for agriculture (mostly rubber and fruit trees) and only 4% is the natural forest cover [36].

The climate conditions in Binh Duong are influenced by tropical monsoons with an average annual temperature and rainfall of 26.5 °C and 1900 mm, respectively, which are suitable for rich vegetation diversity as the soil in this region is fertile [37]. Additionally, the province contains 3 major rivers (Saigon River, Dong Nai River, and Song Be River) and multiple canals.

From 1995 to 2020, Binh Duong Province experienced a significant shift in land use, transitioning from agricultural and unused areas to various other types of land use, as observed by Bui and Mucsi [38]. This was due to the expansion of urbanized areas, mining sites, and artificially irrigated areas into land areas previously used for annual crops or unused croplands. The same study [38] also showed that the expansion of urbanized areas in the province was 65 times between 1995 and 2020. Nevertheless, the root cause of rapid urbanization in the province is the high industrialization rate. Moreover, a recent study by Khuc et al. [39] presented that the concentration of fine particles in the atmosphere in Binh Duong Province has increased in proportion to the expansion of urbanized regions and the concentration of people. Urban flooding is one of the negative effects of infrastructure development without proper planning, and few studies have suggested the implementation of a green infrastructure strategy for managing urban flooding in Binh Duong [40]. Nevertheless, the increase in LST due to rapid urban growth in this rapidly growing metropolitan area is relevant to investigate. The present study considered five highly urbanized districts in southern Binh Duong, namely Thu Dau Mot City, Di An, Thuan An, Ben Cat, and Tan Uyen.

### 2.2. Battambang, Cambodia

Battambang City, situated at 13°06′N and 103°12′E, is the capital of Battambang Province. It ranks as Cambodia’s second-largest city and is situated in the fifth-largest province of the country. Positioned in northwestern Cambodia, the city is approximately 300 kilometers from the national capital, Phnom Penh. According to the Department of Planning [41], Battambang City had a population of 161,072 residents in 2016. The city encompasses a total land area of 115.44 km^2^, with built-up areas constituting 25% of this territory [42]. Agriculture is the primary economic activity in Battambang Province. Carter et al. [43] describe the province as “the rice bowl of Cambodia,” underscoring its significance in the nation’s agricultural sector.

Battambang experiences a tropical monsoon climate characterized by two distinct seasons: the rainy season, spanning from May to October, and the dry season, extending from November to April [44]. The average yearly temperature is 27.7°C, and the area gets about 1,331 mm of rain each year [45].

Han and Lim [46] state that Battambang City lacked an official spatial development plan until 2015. Subsequent to this period, the national government sanctioned the “Land Use Master Plan of Battambang City, 2030.” This Master Plan delineates the spatial organization of the city and outlines the planned long-term purposes of different land-use areas within the governing region. The strategy encompasses comprehensive maps that encompass different sections, including land-utilization zones, transit networks, regions for safeguarding cultural heritage, and initiatives for budget-friendly housing [46].

It is seen that there has been a rapid growth in the population of Battambang City since 2008. While there was only a 0.8% annual growth in population between 2008–2015, it changed to 5.2% between 2015–2016 [41,46]. Research conducted by Sourn et al. [47] revealed that farmland in Battambang province expanded from 44.50% in 1998 to 61.11% in 2008 and continued to grow to 68.40% in 2018. At the same time, the same study estimated that the forest coverage dropped from 29.82% to 6.18% between 1998 and 2018. In addition, Sourn et al. [44] estimated an increase of 4600 ha in built-up areas in Battambang Province, particularly in cities, between 2003 and 2008. Consequently, there will be an increase in the demand for housing and essential urban infrastructure, including roads, drainage and sewage networks, and freshwater provision [46]. Nevertheless, the increase in LST due to rapid urban growth in this rapidly growing metropolitan area is also relevant to investigate.

## 3. Data and methods

LST data, obtained from thermal infrared wavelengths in satellite imagery, has been utilized as a substitute for studying UHIs since the 1970s [20]. Since the launch of Landsat TM imagery in the 1980s, Landsat TM imagery has been used to monitor land use and land cover changes, followed by ASTER imagery, both have thermal data, and several studies on UHI formation expansion based on LST variations have been conducted [4,22]. LST is regarded as a crucial element for determining the Earth’s surface’s radiative burden [48]. As the LST patterns vary from small-scale to large-scale urban areas, LST information can be helpful in the study of urban microclimate [49]. Fonseka et al. [9] noted that the number of developed areas is positively associated with the increase in LST and negatively correlated with other regions, such as vegetation and water. Hully et al. [50] explain that LST can be determined by calculating the emitted surface radiance. This involves correcting sensor radiance data for atmospheric effects and using the inverse Planck function, while considering changes in emissivity.

Despite the advancements in remote sensing technology since the early 1980s, the capabilities of thermal data derived from satellite sensors have been utilized in Vietnam only since the 2010s [4]. Earliest studies, such as Son et al. [21] and Hoan et al. [35] studied UHI developments in Ho Chi Minh City in the south and Hanoi in the north, respectively, using Landsat imagery. Van and Bao [16] used ASTER data instead of Landsat imagery for LST calculation in Ho Chi Minh City.

The current study employed Landsat-8 OLI and TIRS data acquired during the period between 2013 and 2025 to assess the fluctuations in LST in Binh Duong Province in Vietnam and Battambang Province in Cambodia. Only cloud-free images acquired during the dry season were used for the present study. Landsat-8 OLI imagery possesses a spatial resolution of 30m, whereas that of TIRS images is 100m, in addition to the panchromatic imagery with a 15m resolution. Landsat series data in.tiff format can be accessed for free from the United States Geological Survey (USGS) via https://earthexplorer.usgs.gov/. The data contained within the portal has been gathered at regular 16-day intervals and has a resolution of 30 m and WGS1984 coordinate systems [51]. This study utilized only cloud-free, Level 1 Landsat images that were acquired during the study period. Table 1 presents the detailed specifications of the satellite data utilized for each study site. Image processing steps were conducted using Erdas Imagine and ArcGIS software packages.

**Table 1:** Landsat-8 and Landsat-9 data used in the study.

Study area	Satellite	Date of acquisition	Image ID
**Binh Duong**	Landsat 8	21/01/2014	LC08_L1TP_125052_20140121_20200912_02_T1
	Landsat 8	29/03/2015	LC08_L1TP_125052_20150329_20200909_02_T1
	Landsat 8	28/02/2016	LC08_L1TP_125052_20160228_20200907_02_T1
	Landsat 8	14/02/2017	LC08_L1TP_125052_20170214_20200905_02_T1
	Landsat 8	31/10/2018	LC08_L1TP_125052_20181031_20200830_02_T1
	Landsat 8	19/01/2019	LC08_L1TP_125052_20190119_20200829_02_T1
	Landsat 8	23/02/2020	LC08_L1TP_125052_20200223_20200822_02_T1
	Landsat 8	13/03/2021	LC08_L1TP_125052_20210313_20210318_02_T1
	Landsat 8	28/02/2022	LC08_L1TP_125052_20220228_20220309_02_T1
	Landsat 8	19/03/2023	LC08_L1TP_125052_20230319_20230324_02_T1
	Landsat 8	18/02/2024	LC08_L1TP_125052_20240218_20240223_02_T1
	Landsat 8	04/02/2025	LC08_L1TP_125052_20250204_20250208_02_T1
**Battambang**	Landsat 8	09/06/2013	LC08_L1TP_127051_20130609_20200913_02_T1
	Landsat 8	04/02/2014	LC08_L1TP_127051_20140204_20200912_02_T1
	Landsat 8	22/01/2015	LC08_L1TP_127051_20150122_20200910_02_T1
	Landsat 8	14/04/2016	LC08_L1TP_127051_20160414_20200907_02_T1
	Landsat 8	28/02/2017	LC08_L1TP_127051_20170228_20200905_02_T1
	Landsat 8	14/01/2018	LC08_L1TP_127051_20180114_20200902_02_T1
	Landsat 8	25/05/2019	LC08_L1TP_127051_20190525_20200828_02_T1
	Landsat 8	08/03/2020	LC08_L1TP_127051_20200308_20200822_02_T1
	Landsat 8	15/04/2022	LC08_L1TP_127051_20220415_20220420_02_T1
	Landsat 9	25/03/2023	LC09_L1TP_127051_20230325_20230325_02_T1
	Landsat 8	04/04/2024	LC08_L1TP_127051_20240404_20240411_02_T1
	Landsat 9	10/02/2025	LC09_L1TP_127051_20250210_20250210_02_T1

To complement the satellite-based LST analysis, ERA5 reanalysis data were used to assess temporal and spatial variations in near-surface air temperature (2 m) in Battambang (Cambodia) and Binh Duong (Vietnam) from 2013 to 2025. These datasets offer continuous hourly records with a spatial resolution of approximately 31 km × 31 km. The primary source for ERA5 data is the Copernicus Climate Data Store (CDS) and the European Centre for Medium-Range Weather Forecasts (ECMWF), as it’s produced by ECMWF for the European Union’s Copernicus Climate Change Service (C3S). The data is publicly available and can be accessed through CDS, which offers various ways to download and utilize global atmospheric, land-surface, and sea-state parameters [52].

LST variations between 2013 and 2025 in the two study sites (Binh Duong-Vietnam and Battambang-Cambodia) were approximated using the Landsat-8 TIRS data and NDVI from Landsat-8 OLI data as described in Avdan and Jovanovska [53]. Several studies (Guha and Govil 2020) have discussed the relationship between LST and seasonal variations in NDVI [54]. The method by Avdan and Jovanovska [53] is specifically adapted for Landsat OLI/TIRS, which has an improved 12-bit radiometric resolution and directly addresses dual thermal bands (Band 10 and Band 11) and their calibration issues. Moreover, this method uses a single-channel algorithm with correction for atmospheric transmittivity, upwelling radiance, and downwelling radiance. Furthermore, this method integrates emissivity correction using NDVI, which helps to reduce errors related to surface heterogeneity. The 6-step algorithm used for estimating LST in ^o^C [4,23,53] is discussed below:

a Conversion of thermal infrared (TIR) digital number (DN) values into Top of the Atmosphere (TOA) spectral radiance

To begin estimating LST, Band 10 from the TIRS is transformed from Digital Number (DN) values to spectral radiance (R) at the top of the atmosphere using Equation 1.


R = M * D + A − C (1)

Where:

M = band-specific multiplicative rescaling coefficient;

D = Band 10 digital number values of the image;

A = band-specific additive rescaling coefficient;

C = correction factor for Band 10.

The values for M and A are located within the metadata of the Landsat-8 data package. Montanaro et al. [55] advised the utilization of Band 10 TIRS data for the estimation of LST as opposed to Band 11, citing concerns regarding the calibration uncertainty of the latter.

b Conversion of TOA spectral reflectance into at-sensor brightness temperature (BT) in ^**o**^**C**

The second step requires converting the TOA spectral radiance into brightness temperature (T) by applying Equation 2.


$$\text{T }=\;{\text{B}}_{\text{2}}/\text{ ln}[({\text{B}}_{\text{1}}\;/\text{ R})\;+\;\text{1}-\text{ 273}.\text{15}$$ (2)

Where:

B₁ = band-specific thermal conversion coefficient 1 = 774.8853 W/(m^2^.sr.μm);

B₂ = band-specific thermal conversion coefficient 2 = 1321.0789 K

B₁ and B₂ can be found in the metadata of the Landsat-8 data package. To convert these values to brightness temperature in °C, subtract 273.15.

c Normalized Difference Vegetation Index – NDVI

Many studies recognized that LST and land surface characteristics are closely correlated [54]. Due to evaporative cooling, a negative correlation between NDVI and LST is expected [56] (Deng et al. 2018). This negative correlation can be corrected by using NDVI to estimate LST, which can be used to estimate emissivity. NDVI can be estimated by using Equation 3. NDVI is vital for determining land surface emissivity, which is necessary for calculating land surface temperature [54].


NDVI = (B5−B4) / (B5+B4) (3)

B4 = Landsat 8 Band 4 (red)

B5 = Landsat 8 Band 5 (near infrared)

Seasonal variations in the NDVI-LST relationship have been considered as an important variable in the study of mixed urban land surfaces [54]. Generally, the negative correlation between NDVI and LST is stronger in the wet season than in the dry season [57]. Some studies, like Ullah et al. [58], found that NDVI increases with elevation. This information is important for high-altitude urban areas. This observation can be useful in regulating temperature in urban areas by applying a combination of vegetation and height variables, depending on the availability [59].

d Proportion of vegetation (Pv)

To use NDVI for estimating LST, it is crucial to know the amount of vegetation. Deardorff [60] characterized Fv as the proportion of the ground area that is in contact with vegetation, including leaves, stems, and branches, relative to the entire area occupied by vegetation. The proportion of vegetation (Equation 4) is a key biophysical variable related to earth surface processes, important for monitoring biodiversity and modeling climate and weather [61].


$$\text{Fv }=\;[(\text{NDVI }-\;{\text{NDVI}}_{\text{low}})\;/\;({\text{NDVI}}_{\text{high}}\;-\;{\text{NDVI}}_{\text{low}})]²$$ (4)

Where:

NDVI_low_ = the minimum value of NDVI;

NDVI_high_ = the maximum value of NDVI.

e Estimation of emissivity (ε) from the Proportion of vegetation

After estimating the proportion of vegetation, the next step is to calculate the emissivity using Equation 5. A higher proportion of vegetation leads to a higher overall emissivity for the surface.


ϵ = 0.004 + Pv*0.986 (5)

f Estimation of LST from brightness temperature and emissivity

Once you know the brightness temperature and emissivity, you can calculate the LST in degrees Celsius using Equation 6.


LST = (T + (1 + 0.00115 * T/1.138) * Ln(ϵ)) (6)

Where:

T = brightness temperature

ε = emissivity

Air temperature at 2 meters above the surface was obtained from the ERA5 reanalysis, produced by the European Centre for Medium-Range Weather Forecasts (ECMWF). ERA5 provides global climate data at an hourly temporal resolution and a horizontal resolution of approximately 31 km × 31 km. The dataset was accessed for the period from 2013 to 2025, covering the spatial domain between 10.8°S and 13.05°S latitude and 102.8°E to 106.8°E longitude. For each year, hourly temperature values were spatially averaged within the boundaries of each province, as defined by the administrative shapefiles. Daily means were computed by averaging all hourly values for each complete day, followed by monthly and annual means derived from the daily time series. These temporal aggregations were used to characterize the seasonal and interannual variability in near-surface air temperature across the region. The ERA5 dataset is described in detail by Hersbach et al. [30].

## 4. Results and discussion

This study looked at changes in NDVI and LST in two provinces that are growing quickly due to urbanization, one in Cambodia and the other in Vietnam. The NDVI series helped to understand the spatiotemporal changes in land use, whereas the LST series has been employed to explain the influence of land use on urban microclimate.

### 4.1. Trends in NDVI

Typically, NDVI ranges from −1 to +1; a negative value indicates waterbodies and submerged soil, a value above 0 to 0.2 indicates bare soil and built-up areas, and a value above 0.2 indicates all kinds of vegetation. A temporal change in NDVI in agricultural areas indicates seasonal crop cycles, land use changes, or impacts of climate change (e.g., desertification). However, a gradual decrease in NDVI in a time-series map indicates urban sprawl, deforestation, or soil degradation.

**Fig 2:**
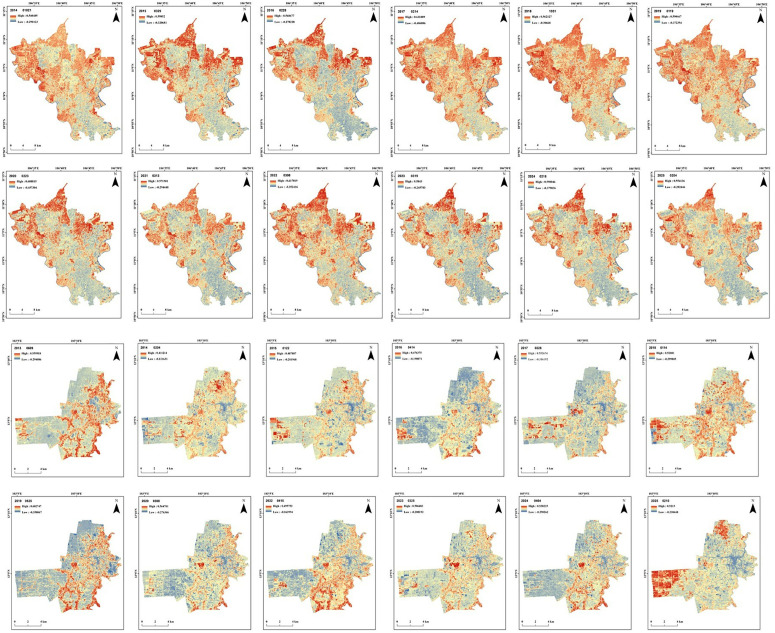
Spatiotemporal variations in NDVI in Binh Duong between 2014-2025(top) and Battambang between 2013-2025 (bottom). (Source: authors).

The time-series map of NDVI in Binh Duong province also showed its fast-growing urban areas during the past decade (Fig 2), particularly in its central and southern regions. The area covered by vegetation (NDVI values more than 0.2) in Binh Duong has reduced from 423 km^2^ to 345 km^2^ during the study period. On the other hand, built-up areas (NDVI values between 0 and 0.2) have increased from 387 km^2^ to 464.3 km^2^.

The observed changes in NDVI agree with previous studies, such as Bui and Musci [38], which estimated a 65-fold expansion of urban areas between 1995 and 2020 (25 years) in Binh Duong province. Another recent study by Huyen et al. [62] also came with similar results on impervious surfaces. The same study [62] highlighted the importance of green areas, including green corridors, natural forests, and planted forests. Compared to Battambang City, where urbanization is concentrated in the eastern areas, the entire southern Binh Duong province underwent urbanization during the study period.

The changes in NDVI patterns over time and space in Battambang city demonstrate the influence of urban development on land use patterns transformations. The swift growth of metropolitan regions in the eastern regions of the study site is visible from the resulting time-series map of NDVI (Fig 2). The differences in NDVI seen on the western side are due to seasonal crops and shifts in land use patterns. The area covered by vegetation in Battambang has reduced from 77.53 km^2^ to 68.87 km^2^ during the study period. On the other hand, built-up areas have increased from 39.2 km^2^ to 47.8 km^2^.

The rapid reduction in NDVI along the eastern side of the study area agrees with a previous study [44] that estimated an exceptional increase in built-up areas from 48 ha to 4698 ha between 1998 and 2018. Unplanned spatial growth of built-up areas is a major cause of rising LST and the development of UHIs [63,64]. In contrast, the forest area has reduced from 358.96 hectares to 74.42 hectares over the same time. Some unusual readings in NDVI in 2023 and 2025 might suggest land being turned into farmland or minor cloud cover affecting the satellite data.

NDVI is highly responsive to plant life cycles and is widely regarded as an excellent measure of vegetation coverage and plant growth. It is also used to study changes in vegetation over various areas and periods. Both the study sites have shown fast-growing urban areas, which make them an ideal location to study the development of urban microclimate. A disadvantage of using NDVI for time-series analysis is that it is typically not stationary, experiencing seasonal variations and both long-term and short-term fluctuations [65]. The present study used images taken during the same season of the year to avoid the effects of seasonality in vegetation from the satellite data. NDVI is important ecologically because it shows changes in biomass. This makes it a valuable tool for understanding the complex relationship between climate change, vegetation distribution patterns, and groundwater availability for woody plants over large areas and time periods [66].

### 4.2. Trends in land surface temperatures

The spatiotemporal changes in LST indicated both a decline in vegetation coverage and a rise in impervious surfaces in both study areas. A color scale from light to dark orange has been used to indicate LST (darker areas indicate a higher LST compared to lighter ones). Urban and densely populated areas consistently exhibit higher LST values throughout the study period, likely due to urban heat island formation.

**Fig 3:**
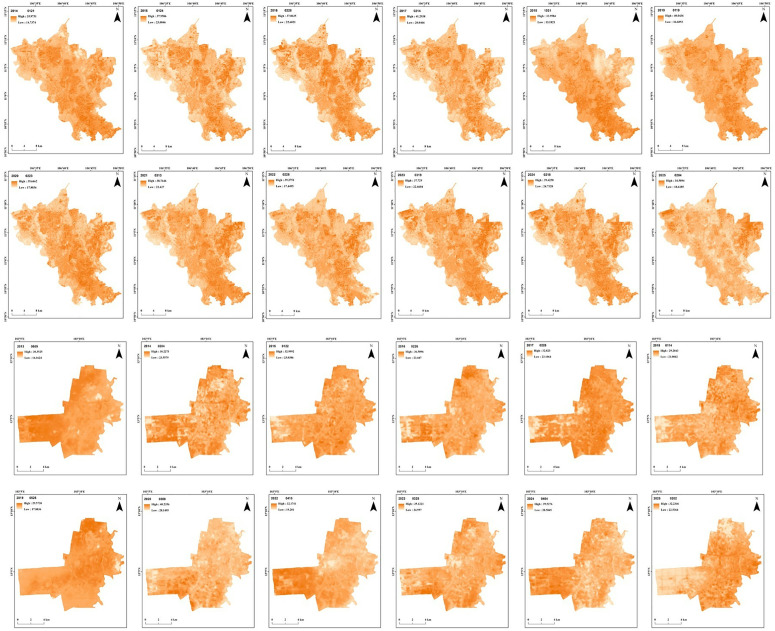
Spatiotemporal variations in land surface temperatures in Binh Duong between 2014 and 2025 (top) and in Battambang between 2014 and 2025 (bottom). (Source: authors).

The LST series between 2014 and 2025 in Binh Duong (Fig 3) indicated a gradual increase in land surface temperatures during the study period. The minimum estimated LST in Binh Duong varied between 13.2 °C to 24.73 °C (and increased 0.26 °C per year), whereas the maximum LST varied between 34.6 °C to 41.3 °C (and increased 0.024 °C per year). Spatial patterns indicate a consistently higher LST in urban areas across the years considered, indicating the formation of UHIs. Even though there is a fluctuation in LST, the general trend is an increase in maximum LST, likely due to urbanization, vegetation loss, and climate change.

The LST series in Battambang (Fig 3) indicated a rise in LST, particularly after 2020, and the central region of the study area is consistently warmer due to the development of urban areas. The minimum estimated LST in Battambang varied between 16.1 °C and 28.58 °C (and increased 0.35 °C per year), whereas the maximum LST varied between 29.2 °C to 40.23 °C (and increased 0.35 °C per year). There has been a notable increase in minimum LST since 2017, probably indicating warmer nights with less cooling. The LST range became narrow after 2020, indicating an overall warming with less variability.

In general, both the study sites have shown an overall increase in LST during the study period. Rapid urbanization causes substantial adverse impacts on the environment [67]. In study areas, where such an impact is worrying due to urban growth, it resulted in increased LST. The continuous growth of impervious surfaces leads to an increase in LST in cities [64]. Furthermore, LST plays a substantial role in investigating the land surface energy budget [67]. By evaluating and observing LST changes, the authorities can use this information as a vital parameter for identifying temperature-related problems in urban areas because spatial urban growth and its impact on LST are a high-priority environmental issue for urban policy [19].

### 4.3. Trends in near-surface air temperature (ERA5)

**Fig 4:**
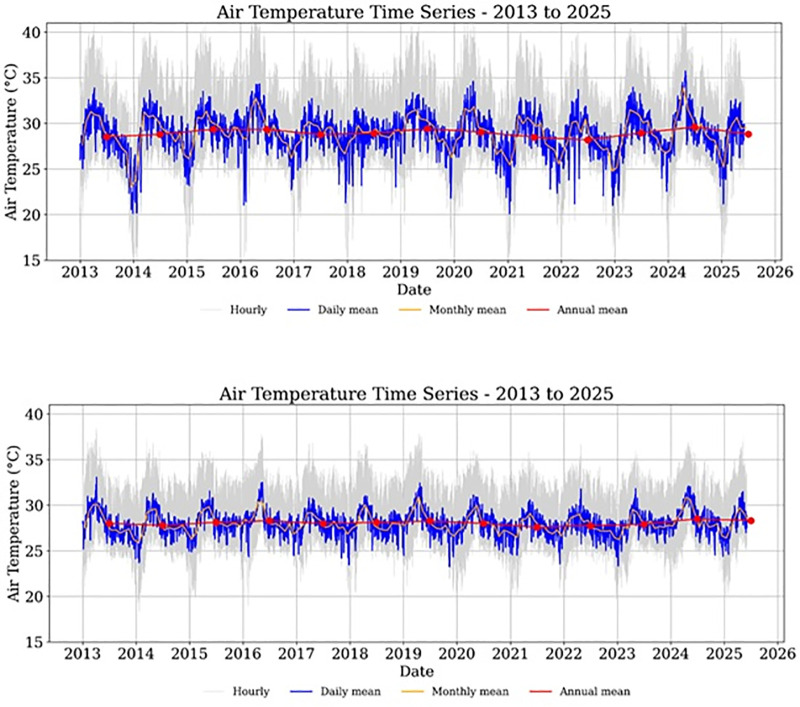
Time series of near-surface air temperature (2 m, ERA5) from 2013 to 2025 for Battambang (top) and Binh Duong (bottom). Hourly data are shown in gray, daily means in blue, monthly means in orange, and annual means in red with linear trend.

Time series of air temperature (Fig 4) reveal distinct seasonal cycles and interannual variability in both study sites. In general, the hourly and daily data show clear seasonal oscillations, while the monthly and annual aggregates provide insight into long-term patterns associated with urban development and land cover change.

It is visible from the graphs (Fig 4) that the mean annual temperature in Battambang was ~ 28 °C in 2013, which gradually increased to ~29 °C in 2025, suggesting a ~ 1 °C increase in 12 years. Strong inter-annual variability but with a clear warning signal was observed. Seasonal cycle remains consistent, but peaks seem slightly higher in later years. The warming trend is modest but noticeable, reflecting possible variations in urban microclimate. Unlike Battambang, the graph representing Binh Duong shows a weaker upward trend in the annual mean (~0.34 °C increase in the study period). Here, seasonality dominates, and the annual mean shows a mild warming trend. The long-term mean temperature stays close to 28–29°C, suggesting a relatively stable climate with slight warming.

**Fig 5:**
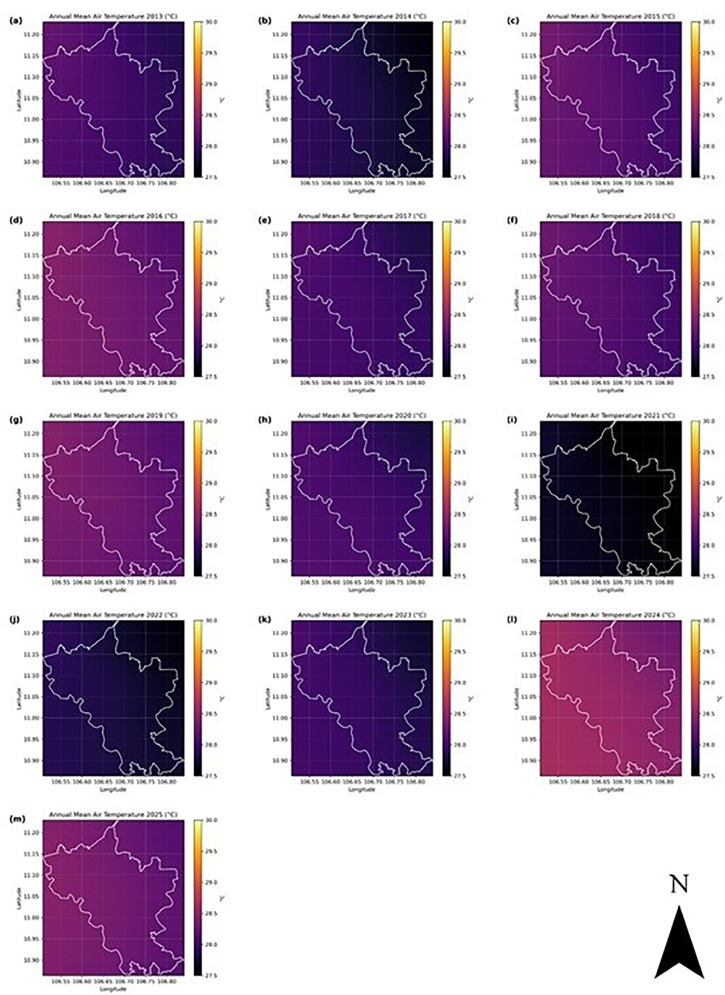
Annual mean air temperature (ERA5, °C) in Binh Duong, Vietnam, from 2013 to 2025. The white polygon shows the administrative boundary of the province.

**Fig 6:**
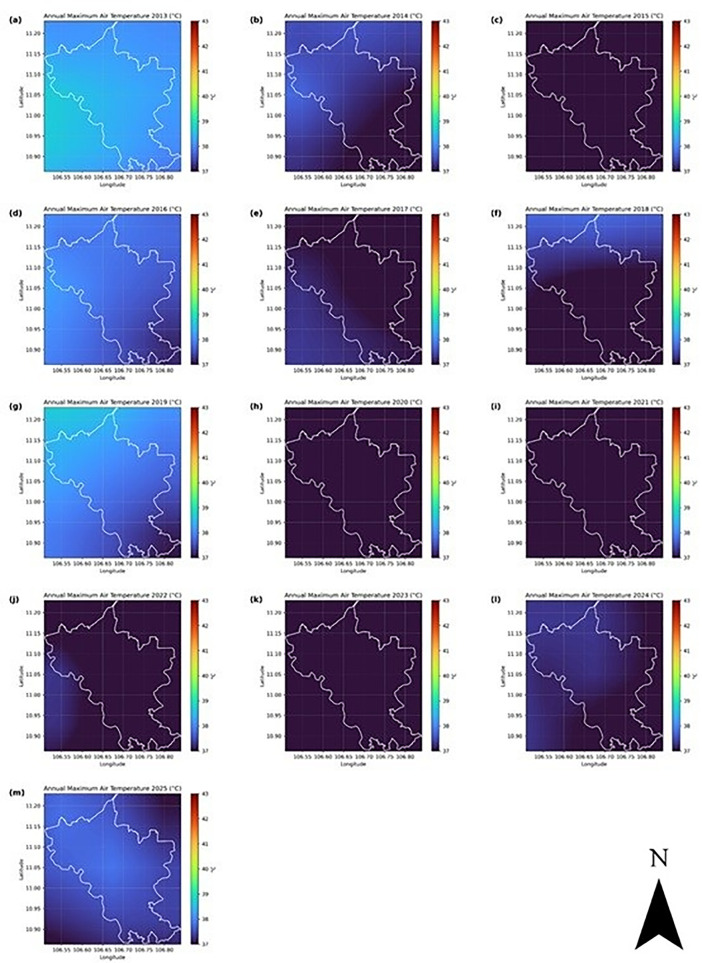
Annual maximum air temperature (ERA5, °C) in Binh Duong, Vietnam, from 2013 to 2025. The white polygon shows the administrative boundary of the province.

In Binh Duong, the annual mean air temperature remained relatively stable around 29 °C, with notable seasonal amplitude and limited variability in the long-term trend. The spatial maps of annual mean and maximum temperatures (Figs 5 and 6) show consistently higher values in the southern region of the province, especially in urban districts such as Thu Dau Mot, Di An, and Thuan An. These spatial patterns align with areas of intense urbanization and industrialization described in Section 2.1 and are indicative of the establishment of urban heat island (UHI) conditions. The maximum air temperature regularly exceeds 40 °C in some years, further supporting this hypothesis.

The temporal trends in the annual mean air temperatures in Binh Duong indicated a gradual (but not linear) warming trend during the period analyzed. Western and northern areas appear slightly cooler (darker shades), while central and southern zones tend to be warmer (yellowish to reddish tones). The spatial gradient is persistent across years, suggesting the influence of topography, land cover changes (built-up areas, impervious surfaces), or coastal effects. The land cover changes based on NDVI data (Fig 2) and LST changes from satellite imagery (Fig 3) support this observation. The variation is typically within ~1.0–1.5 °C across the region each year. The years 2014 and 2021 were exceptionally cooler (close to 28 °C), and 2024–2025 were warmer (mean temperatures above 29 °C), and these anomalies might correspond to regional climate events, such as El Niño/La Niña phases. The relatively small temperature range (approximately 2.5°C) across the entire time series indicates a climatologically stable region, yet the clear year-to-year variations highlight the importance of monitoring regional temperature trends for understanding local climate dynamics and potential impacts on ecosystem functioning and human activities. The annual maximum air temperature series reveals a nonlinear pattern with early (2013–2015) and late (2025) warmer peaks, separated by a cooling anomaly (2017–2022). The spatial pattern is consistent, with central/eastern zones hotter than western/northern areas. While mean temperatures steadily rose, maximum extremes fluctuated but are trending back upward, signaling possible intensification of extreme heat events in the future.

**Fig 7:**
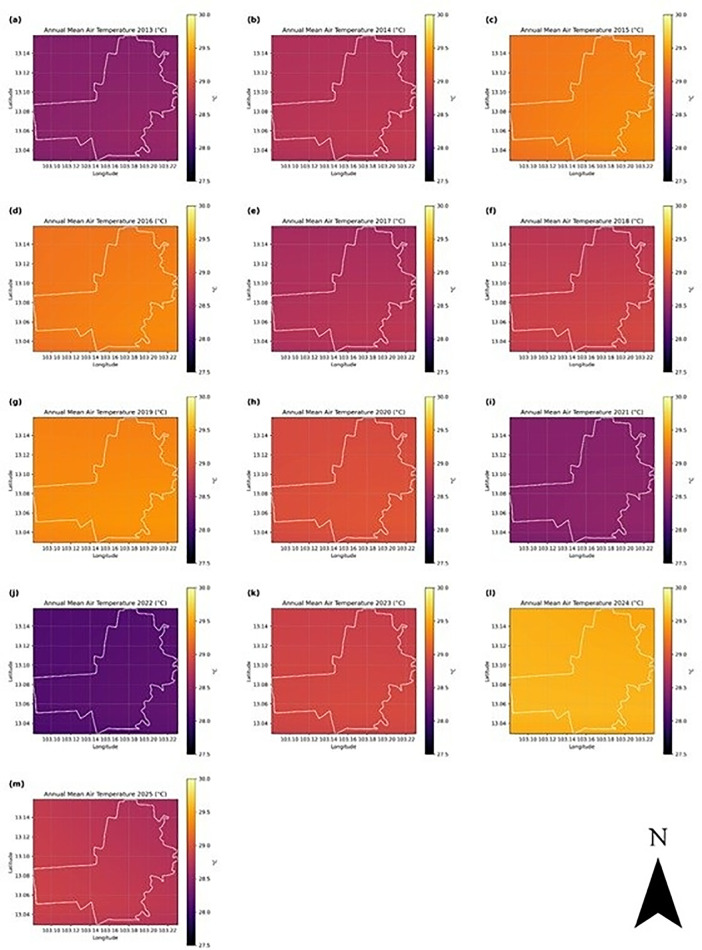
Annual mean air temperature (ERA5, °C) in Battambang, Cambodia, from 2013 to 2025. The white polygon shows the administrative boundary of the province.

**Fig 8:**
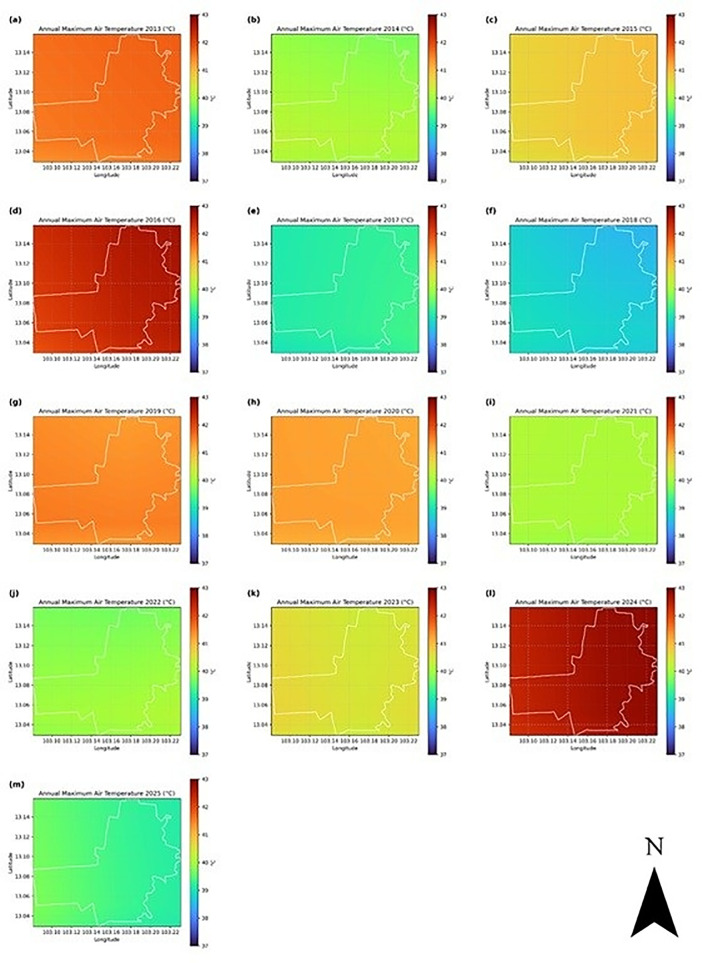
Annual maximum air temperature (ERA5, °C) in Battambang, Cambodia, from 2013 to 2025. The white polygon shows the administrative boundary of the province.

In contrast, Battambang Province exhibits a more pronounced temporal pattern, with increasing fluctuations in daily and monthly means observed particularly after 2020 (Fig 4). Despite interannual variability, the annual mean air temperature hovered around 28.5 °C during most of the period. Spatially, the maps of annual mean and maximum temperature (Figs 7 and 8) highlight persistent warming in the central and eastern parts of the province, regions undergoing accelerated urban development, as documented in Section 2.2. This observation is consistent with the reported increase in minimum LST in Battambang after 2017 and supports the inference of UHI intensification due to urban sprawl and vegetation loss.

In the Battambang region (Fig 7), across all years, annual mean air temperature gradients are visible along longitude (west-east direction). The time-series maps demonstrate considerable interannual variability in temperature patterns across the study period. The region experiences a temperature range of approximately 2.5°C, from 27.5°C to 30.0°C. Warmer zones appear consistently in the eastern and southeastern areas, while the northwestern areas stay relatively cooler. The spatial pattern remains stable, suggesting that local topography or land cover (including built-up area expansion as evident from time-series NDVI maps in Fig 2) strongly influences mean temperature distribution. While the time series is relatively short (13 years), the overall trajectory suggests gradual warming with short-term variability. The pronounced year-to-year temperature fluctuations suggest the region is subject to significant climate variability, potentially influenced by larger-scale climate phenomena such as El Niño-Southern Oscillation (ENSO), monsoon patterns, or other regional climate drivers. The year-to-year fluctuations (~1.5 °C range) are larger than the long-term warming (~0.6 °C), which is typical for regional climate influenced by ENSO and local variability. The region is warming at an estimated +0.5 °C per decade (2013–2025), but with strong interannual variability driven by short-term climate events. The annual maximum air temperature (Fig 8) shows that the central and eastern parts of the region tend to show higher maximum temperatures in many years. This may indicate geographic or land-use influences (urban heat islands or elevation), which are evident from NDVI (Fig 2) and LST (Fig 3) time-series.

Overall, the analysis of ERA5 near-surface air temperature data corroborates the findings based on LST and NDVI, confirming that both study areas experienced localized warming trends attributable to urban expansion. The integration of reanalysis-based air temperature data provides an additional line of evidence for understanding the urban microclimate dynamics and enhances the robustness of UHI detection at the regional scale.

### 4.4. Limitations of the data and methods used in the present study

Despite the widespread use of Landsat data for UHI and urbanization studies, several limitations need to be considered. Landsat has a 16-day revisit cycle (8 days if combining Landsat 8 and Landsat 9), which is too coarse for monitoring rapid temperature dynamics such as diurnal or short-term weather changes. This limits its usefulness in studying transient phenomena, such as heatwaves, irrigation cycles, or daily urban thermal variations. Landsat’s thermal infrared (TIR) bands have a 100 m spatial resolution (resampled to 30 m), which is coarser than its visible/NIR bands. This may cause mixed pixel effects, especially in heterogeneous environments (urban mosaics, fragmented agricultural fields, water–land boundaries). Accurate LST calculation depends on surface emissivity, which varies with land cover type (e.g., soil, vegetation, impervious surfaces, water) [68]. Misclassification or generalized emissivity assumptions can introduce bias in LST retrieval [69]. Landsat TIR cannot penetrate clouds, and cloud cover masks large portions of data in tropical and humid regions (which is why we have used cloud-free images during the same season for the present study). Even thin cirrus clouds can distort thermal signals, reducing data availability and reliability. Landsat captures data at around 10:00 a.m. local solar time, missing nighttime or afternoon thermal conditions. This single-time snapshot cannot represent full diurnal LST variations, limiting applications such as urban heat island intensity comparisons between day and night. Common LST retrieval methods each have assumptions that may not be held under all conditions [70]. Errors increase over complex terrains, coastal areas, or semi-arid zones where emissivity and atmospheric effects are highly variable [70].

Even though ERA5 is currently one of the most widely used global reanalysis datasets, with hourly estimates of atmospheric, land, and ocean variables at ~31 km resolution, it also has a few limitations. Despite being finer than ERA-Interim (~80 km), ERA5 is still coarse to capture urban microclimates, mountain–valley temperature gradients, and coastal effects [71]. ERA5 underestimates daily maximum temperatures and overestimates daily minimum temperatures, leading to a narrower diurnal temperature range. Cold biases are often observed in high-latitude and snow/ice regions, while warm biases may occur in arid/semi-arid regions [72]. In mountainous areas, the coarse grid and model topography cause mismatches with station elevation, leading to significant biases [71]. ERA5 tends to smooth extremes (e.g., heatwaves, cold spells, frost events) due to grid averaging and model physics. Sub-daily extremes and short-duration heatwaves may be underestimated [73]. ERA5 relies on a combination of satellite, radiosonde, and surface station data [30]. In regions with sparse observations (e.g., parts of Africa, South America, polar regions, oceans), the accuracy is reduced, and temperature estimates lean more heavily on model physics [74]. Although ERA5 is more consistent than ERA-Interim, changes in observing systems (e.g., new satellite instruments introduced over time) may still cause subtle artificial trends in long-term air temperature series. Surface temperature estimates depend on land surface models, which may have errors in soil moisture, vegetation cover, or snow cover representation [72]. These errors propagate into 2 m air temperature estimates, especially in regions with strong land–atmosphere feedback. ERA5 provides data at hourly intervals with multiple ensemble members, leading to large data volumes. This makes downloading, processing, and storing ERA5 data resource-intensive, especially for long time periods or global studies. While ERA5 is excellent for global and regional climate studies, it cannot substitute in-situ measurements for applications requiring local accuracy (e.g., agriculture, hydrology, urban climate adaptation). Nevertheless, ERA5 datasets offer a cost-effective solution to climate data analysis.

## 5. Conclusions and recommendations

Fast urban growth leads to negative environmental effects, such as increased LST and the creation of UHI. These areas become warmer than their surroundings because they absorb sunlight and release it as heat. This study investigated the LST changes in two provinces undergoing swift urbanization, Vietnam (Binh Duong) and Cambodia (Battambang), between 2013 and 2025. LST variations are crucial for understanding how human activities affect urban areas, contribute to the creation of UHIs, and influence the urban microclimate.

The time-series analysis of NDVI indicated urban areas’ expansion and vegetation reduction in both Battambang and Binh Duong. While the rapid expansion of metropolitan areas in Battambang focused on its eastern region, the impervious surfaces, including asphalt roads, concrete, and rooftops, have rapidly expanded in the entire southern Binh Duong province. The spatiotemporal changes in LST indicated a decline in vegetation coverage and an increase in the area covered by impervious surfaces in Binh Duong and Battambang. The minimum estimated LST in Battambang has increased about 0.35 °C per year, whereas the maximum LST has increased about 0.36 °C per year. The LST in southern Binh Duong increased gradually between 2014 and 2025, primarily due to rapid urbanization and vegetation loss. The minimum estimated LST in Binh Duong has increased about 0.26 °C per year, whereas the maximum LST has increased about 0.024 °C per year. The findings reveal that LSTs have risen over the years due to the creation of UHIs. These changes highlight the negative effects of quick urbanization and the growth of impervious surfaces, despite also contributing to financial stability and economic growth.

Future research should combine Landsat and ERA5 with other satellite missions such as MODIS, Sentinel-3 SLSTR, or VIIRS to overcome temporal and spatial limitations. Multi-sensor fusion and downscaling techniques could provide higher temporal resolution while maintaining Landsat’s spatial detail. Other robust retrieval methods, such as split-window algorithms, radiative transfer models, or machine learning approaches, can minimize emissivity and atmospheric correction uncertainties. Local calibration using ground-based meteorological and flux tower data would improve retrieval accuracy in complex tropical environments. Extend analysis beyond LST patterns to assess urban heat exposure, health impacts, and socio-economic vulnerabilities. Findings could be directly linked to urban planning and heat mitigation policies in rapidly growing cities of Vietnam and Cambodia. Since ERA5 has coarse resolution, statistical or dynamical downscaling should be applied to produce city-scale climate datasets. Bias correction using local station data can improve ERA5’s applicability for urban climate adaptation studies. Integrating ERA5’s hourly air temperature with thermal data from geostationary satellites (e.g., Himawari-8) could capture day–night and seasonal LST dynamics.

Future work should combine LST with land use/land cover (LULC) maps, population density, and infrastructure growth data to better explain drivers of temperature evolution. This would enhance understanding of how urbanization intensity relates to LST change. Incorporate regional climate model (RCM) projections to assess how ongoing urban expansion in Southeast Asia may interact with global warming trends. Scenario-based studies could provide valuable foresight for sustainable urban design. Research should not only quantify LST but also assess heat vulnerability at community levels, particularly in informal settlements. Linking LST studies with green infrastructure, water-sensitive urban design, and policy-driven cooling strategies would increase societal relevance. Expanding the scope beyond Vietnam and Cambodia to include other rapidly urbanizing Southeast Asian cities (Bangkok, Manila, Jakarta) could establish a regional comparative framework, offering lessons transferable across the Mekong and ASEAN region.

## References

[1] Li Z-L, Tang B-H, Wu H, Ren H, Yan G, Wan Z, et al. Satellite-derived land surface temperature: current status and perspectives. Remote Sens Environ. 2013;131:14–37. doi: 10.1016/j.rse.2012.12.008

[2] Hereher ME. Time series trends of land surface temperatures in Egypt: a signal for global warming. Environ Earth Sci. 2016;75(17). doi: 10.1007/s12665-016-6024-4

[3] Tayyebi A, Shafizadeh-Moghadam H, Tayyebi AH. Analyzing long-term spatio-temporal patterns of land surface temperature in response to rapid urbanization in the mega-city of Tehran. Land Use Policy. 2018;71:459–69. doi: 10.1016/j.landusepol.2017.11.023

[4] Veettil BK, Puri V, Van DD, Quang NX. Variations in land surface temperatures in small-scale urban areas in Vietnam during Covid-19 restrictions: case studies from Da Nang, Hue and Vinh City. Environ Monit Assess. 2023;195(7):822. doi: 10.1007/s10661-023-11332-4 37291411 PMC10250182

[5] Argüeso D, Evans JP, Fita L, Bormann KJ. Temperature response to future urbanization and climate change. Clim Dyn. 2013;42(7–8):2183–99. doi: 10.1007/s00382-013-1789-6

[6] Zeleňáková M, Purcz P, Hlavatá H, Blišťan P. Climate change in urban versus rural areas. Procedia Eng. 2015;119:1171–80. doi: 10.1016/j.proeng.2015.08.968

[7] Ojeh V, Balogun A, Okhimamhe A. Urban-rural temperature differences in Lagos. Climate. 2016;4(2):29. doi: 10.3390/cli4020029

[8] Peng S, Piao S, Ciais P, Friedlingstein P, Ottle C, Bréon F-M, et al. Surface urban heat island across 419 global big cities. Environ Sci Technol. 2012;46(2):696–703. doi: 10.1021/es2030438 22142232

[9] Fonseka HPU, Zhang H, Sun Y, Su H, Lin H, Lin Y. Urbanization and its impacts on land surface temperature in Colombo Metropolitan Area, Sri Lanka, from 1988 to 2016. Remote Sens. 2019;11(8):957. doi: 10.3390/rs11080957

[10] Said ZM, Dindar S. Key challenges and strategies in the evaluation of sustainable urban regeneration projects: insights from a systematic literature review. Sustainability. 2024;16(22):9903. doi: 10.3390/su16229903

[11] Guo Z, Wang SD, Cheng MM, Shu Y. Assess the effect of different degrees of urbanization on land surface temperature using remote sensing images. Procedia Environ Sci. 2012;13:935–42. doi: 10.1016/j.proenv.2012.01.087

[12] Veettil BK, Grondona AEB. Vegetation changes and formation of small-scale urban heat islands in three populated districts of Kerala State, India. Acta Geophys. 2018;66(5):1063–72. doi: 10.1007/s11600-018-0189-z

[13] Dindar S. A systematic review of urban regeneration’s impact on sustainable transport: traffic dynamics, policy responses, and environmental implications. Sustain Dev. 2025. doi: 10.1002/sd.70007

[14] Maheshwari B, Pinto U, Akbar S, Fahey P. Is urbanisation also the culprit of climate change? – Evidence from Australian cities. Urban Climate. 2020;31:100581. doi: 10.1016/j.uclim.2020.100581

[15] Voogt JA, Oke TR. Thermal remote sensing of urban climates. Remote Sens Environ. 2003;86(3):370–84. doi: 10.1016/s0034-4257(03)00079-8

[16] Thi Van T, Duong Xuan Bao H. Study of the impact of urban development on surface temperature using remote sensing in Ho Chi Minh City, Southern Vietnam. Geogr Res. 2010;48(1):86–96. doi: 10.1111/j.1745-5871.2009.00607.x

[17] Cetin M, Ozenen Kavlak M, Senyel Kurkcuoglu MA, Bilge Ozturk G, Cabuk SN, Cabuk A. Determination of land surface temperature and urban heat island effects with remote sensing capabilities: the case of Kayseri, Türkiye. Nat Hazards. 2024;120(6):5509–36. doi: 10.1007/s11069-024-06431-5

[18] Singh R, Grover A. Spatial correlations of changing land use, surface temperature (UHI) and NDVI in Delhi using Landsat satellite images. Urban Development Challenges, Risks and Resilience in Asian Mega Cities. Springer; 2015: 83–97.

[19] Rahman MdM, Avtar R, Yunus AP, Dou J, Misra P, Takeuchi W, et al. Monitoring effect of spatial growth on land surface temperature in Dhaka. Remote Sens. 2020;12(7):1191. doi: 10.3390/rs12071191

[20] Rao PK. Remote sensing of urban “heat islands” from an environmental satellite. Bull Am Meteorol Soc. 1972;53:647–8.

[21] Son N-T, Chen C-F, Chen C-R, Thanh B-X, Vuong T-H. Assessment of urbanization and urban heat islands in Ho Chi Minh City, Vietnam using Landsat data. Sustain Cities Soc. 2017;30:150–61. doi: 10.1016/j.scs.2017.01.009

[22] Zhou D, Xiao J, Bonafoni S, Berger C, Deilami K, Zhou Y, et al. Satellite remote sensing of surface urban heat islands: progress, challenges, and perspectives. Remote Sens. 2018;11(1):48. doi: 10.3390/rs11010048

[23] Veettil BK, Van DD. Did the Covid-19 restrictions influence land surface temperatures in Southeast Asia? A study from Ho Chi Minh City, Vietnam. Environ Sci Pollut Res Int. 2023;30(25):66812–21. doi: 10.1007/s11356-023-26892-8 37186185 PMC10130310

[24] Khan D, Bano S, Khan N. Spatio-temporal analysis of urbanization effects: unravelling land use and land cover dynamics and their influence on land surface temperature in Aligarh City. Geol Ecol Landscapes. 2024:1–25. doi: 10.1080/24749508.2024.2409488

[25] Pandey A, Mondal A, Guha S, Upadhyay PK, Rashmi, Kundu S. Comparing the seasonal relationship of land surface temperature with vegetation indices and other land surface indices. Geol Ecol Landscapes. 2024;:1–17. doi: 10.1080/24749508.2024.2392391

[26] Chapman L, Azevedo JA, Prieto-Lopez T. Urban heat & critical infrastructure networks: a viewpoint. Urban Clim. 2013;3:7–12. doi: 10.1016/j.uclim.2013.04.001

[27] Amnuaylojaroen T. Intensification of heat extremes in Southeast Asia: spatial–temporal analysis of temperature trends and heat events (1940–2023). Intl J Climatol. 2025;45(10). doi: 10.1002/joc.8907

[28] Estrada F, Perron P. Disentangling the trend in the warming of urban areas into global and local factors. Ann N Y Acad Sci. 2021;1504(1):230–46. doi: 10.1111/nyas.14691 34529855 PMC9290917

[29] Mahmoodi S, Dutta K, Basu D, Agrawal S. Understanding link between land surface temperature and landscape heterogeneity: a spatio-temporal and inter-seasonal variability study on Kabul city, Afghanistan. ISPRS Ann Photogramm Remote Sens Spatial Inf Sci. 2019;IV-5/W2:57–65. doi: 10.5194/isprs-annals-iv-5-w2-57-2019

[30] Hersbach H, Bell B, Berrisford P, Hirahara S, Horányi A, Muñoz‐Sabater J, et al. The ERA5 global reanalysis. Quart J Royal Meteoro Soc. 2020;146(730):1999–2049. doi: 10.1002/qj.3803

[31] Arouri M, Ben Youssef A, Nguyen C. Does urbanization reduce rural poverty? Evidence from Vietnam. Econ Modell. 2017;60:253–70. doi: 10.1016/j.econmod.2016.09.022

[32] Pham Thi N, Kappas M, Wyss D. Benefits and constraints of the agricultural land acquisition for urbanization for household gender equality in affected rural communes: a case study in Huong Thuy Town, Thua Thien Hue Province, Vietnam. Land. 2020;9(8):249. doi: 10.3390/land9080249

[33] Le Hung T, Zablotskii VR, Zenkov IV, Vu DT, Dao KH. Relationship between the land surface temperature and land cover types, a case study in Hanoi City, Vietnam. Izv Atmos Ocean Phys. 2022;58(9):1111–20. doi: 10.1134/s0001433822090067

[34] Mohiuddin G, Mund J-P. Spatiotemporal analysis of land surface temperature in response to land use and land cover changes: a remote sensing approach. Remote Sens. 2024;16(7):1286. doi: 10.3390/rs16071286

[35] Thanh Hoan N, Liou Y-A, Nguyen K-A, Sharma RC, Tran D-P, Liou C-L, et al. Assessing the effects of land-use types in surface urban heat islands for developing comfortable living in Hanoi City. Remote Sens. 2018;10(12):1965. doi: 10.3390/rs10121965

[36] Thuy VT, Huyen NT, Tu LH, Loi NK. Status of bamboos in Binh Duong province, Vietnam: distribution, species diversity, conservation and utilization. Trees, Forests People. 2021;6:100137. doi: 10.1016/j.tfp.2021.100137

[37] Binh Duong People’s Committee. Natural conditions. Internet portal of Binh Duong People’s Committee. 2018 [Accessed 2025 January 10]. https://eng.binhduong.gov.vn/Lists/GioiThieu/DispForm.aspx?ID=16&InitialTabId=Ribbon

[38] Bui DH, Mucsi L. Land-use change and urban expansion in Binh Duong province, Vietnam, from 1995 to 2020. Geocarto Int. 2022;37(27):17096–118. doi: 10.1080/10106049.2022.2123564

[39] Khuc TD, Nguyen LQ, Tran DT, Anh Tran V, Nguyen QN, Truong XQ, et al. Assessing the effect of open-pit mining activities and urbanization on fine particulate matter concentration by using remote sensing imagery: a case study in Binh Duong Province, Vietnam. In: Nguyen LQ, Bui LK, Bui XN, Tran HT, eds. Environmental science and engineering. Springer International Publishing; 2023: 75–94. doi: 10.1007/978-3-031-20463-0_5

[40] An TNT, Huong LTT. Review of a green infrastructure approach in urban flood management and its possible application in Binh Duong Province. In: Mookherjee D, Pomeroy GM, Huong LTT, eds. Advances in 21st century human settlements. Springer Nature Singapore; 2023: 491–503. doi: 10.1007/978-981-19-8726-7_29

[41] Department of Planning. The 2017 social economics: battambang city. Battambang: Department of Planning; 2017.

[42] Battambang Municipality. Technical report on the land use master plan for Battambang Municipality. Battambang Municipality; 2015.

[43] Carter R, Grayson G, Hewitt J. Strategic guidelines for heritage tourism in Battambang Province, Cambodia. 2016. https://www.researchgate.net/publication/303566295_Strategic_Guidelines_for_Heritage_Tourism_in_Battambang_Province_Cambodia

[44] Sourn T, Pok S, Chou P, Nut N, Theng D, Rath P, et al. Evaluation of land use and land cover change and its drivers in Battambang Province, Cambodia from 1998 to 2018. Sustainability. 2021;13(20):11170. doi: 10.3390/su132011170

[45] Nut N, Mihara M, Jeong J, Ngo B, Sigua G, Prasad PVV, et al. Land use and land cover changes and its impact on soil erosion in stung sangkae catchment of Cambodia. Sustainability. 2021;13(16):9276. doi: 10.3390/su13169276

[46] Han SS, Lim Y. Battambang City, Cambodia: From a small colonial settlement to an emerging regional centre. Cities. 2019;87:205–20. doi: 10.1016/j.cities.2018.10.003

[47] Sourn T, Pok S, Chou P, Nut N, Theng D, Prasad PVV. Assessment of land use and land cover changes on soil erosion using remote sensing, GIS and RUSLE model: a case study of Battambang Province, Cambodia. Sustainability. 2022;14(7):4066. doi: 10.3390/su14074066

[48] de Almeida CR, Teodoro AC, Gonçalves A. Study of the Urban Heat Island (UHI) using remote sensing data/techniques: a systematic review. Environments. 2021;8(10):105. doi: 10.3390/environments8100105

[49] Heinl M, Hammerle A, Tappeiner U, Leitinger G. Determinants of urban–rural land surface temperature differences – A landscape scale perspective. Landscape Urban Plann. 2015;134:33–42. doi: 10.1016/j.landurbplan.2014.10.003

[50] Hulley GC, Ghent D, Göttsche FM, Guillevic PC, Mildrexler DJ, Coll C. Land surface temperature. Taking the temperature of the earth. Elsevier; 2019: 57–127. doi: 10.1016/b978-0-12-814458-9.00003-4

[51] Qadri SMT, Hamdan A, Raj V, Ehsan M, Shamsuddin N, Hakimi MH, et al. Assessment of land surface temperature from the Indian Cities of Ranchi and Dhanbad during COVID-19 lockdown: implications on the urban climatology. Sustainability. 2023;15(17):12961. doi: 10.3390/su151712961

[52] Copernicus Climate Change Service. ERA5 hourly data on single levels from 1940 to present. Copernicus Climate Change Service (C3S) Climate Data Store (CDS); 2023. doi: 10.24381/cds.adbb2d47

[53] Avdan U, Jovanovska G. Algorithm for automated mapping of land surface temperature using LANDSAT 8 satellite data. J Sens. 2016;2016:1–8. doi: 10.1155/2016/1480307

[54] Guha S, Govil H. Land surface temperature and normalized difference vegetation index relationship: a seasonal study on a tropical city. SN Appl Sci. 2020;2(10). doi: 10.1007/s42452-020-03458-8

[55] Montanaro M, Lunsford A, Tesfaye Z, Wenny B, Reuter D. Radiometric calibration methodology of the Landsat 8 thermal infrared sensor. Remote Sens. 2014;6(9):8803–21. doi: 10.3390/rs6098803

[56] Deng Y, Wang S, Bai X, Tian Y, Wu L, Xiao J, et al. Relationship among land surface temperature and LUCC, NDVI in typical karst area. Sci Rep. 2018;8(1):641. doi: 10.1038/s41598-017-19088-x 29330526 PMC5766486

[57] Guha S, Govil H, Gill N, Dey A. Analytical study on the relationship between land surface temperature and land use/land cover indices. Ann GIS. 2020;26(2):201–16. doi: 10.1080/19475683.2020.1754291

[58] Ullah W, Ahmad K, Ullah S, Tahir AA, Javed MF, Nazir A, et al. Analysis of the relationship among land surface temperature (LST), land use land cover (LULC), and normalized difference vegetation index (NDVI) with topographic elements in the lower Himalayan region. Heliyon. 2023;9(2):e13322. doi: 10.1016/j.heliyon.2023.e13322 36825192 PMC9942242

[59] Alexander C. Influence of the proportion, height and proximity of vegetation and buildings on urban land surface temperature. Int J Appl Earth Observation Geoinform. 2021;95:102265. doi: 10.1016/j.jag.2020.102265

[60] Deardorff JW. Efficient prediction of ground surface temperature and moisture, with inclusion of a layer of vegetation. J Geophys Res. 1978;83(C4):1889–903. doi: 10.1029/jc083ic04p01889

[61] Gutman G, Ignatov A. The derivation of the green vegetation fraction from NOAA/AVHRR data for use in numerical weather prediction models. Int J Remote Sens. 1998;19(8):1533–43. doi: 10.1080/014311698215333

[62] Thi Huyen N, Tu LH, Ngoc Han LT, Thi Thuy V, Dong Phuong DN, Kim Loi N. Assessing vegetation cover change using remote sensing: case study at Binh Duong Province, Vietnam. App Envi Res. 2022;:17–32. doi: 10.35762/aer.2022.44.3.2

[63] Masroor M, Avtar R, Sajjad H, Choudhari P, Kulimushi LC, Khedher KM, et al. Assessing the influence of land use/land cover alteration on climate variability: an analysis in the aurangabad district of Maharashtra State, India. Sustainability. 2022;14(2):642. doi: 10.3390/su14020642

[64] Mallick SK, Sahu N, Das P, Maity B, Varun A, Kumar A, et al. Impact of urban growth in Delhi and It’s Peri-urban environment on urban heat exposure. Urban Clim. 2024;56:102010. doi: 10.1016/j.uclim.2024.102010

[65] Martínez B, Gilabert MA. Vegetation dynamics from NDVI time series analysis using the wavelet transform. Remote Sens Environ. 2009;113(9):1823–42. doi: 10.1016/j.rse.2009.04.016

[66] Aguilar C, Zinnert JC, Polo MJ, Young DR. NDVI as an indicator for changes in water availability to woody vegetation. Ecol Indicators. 2012;23:290–300. doi: 10.1016/j.ecolind.2012.04.008

[67] Kanga S, Meraj G, Johnson BA, Singh SK, Pv MN, Farooq M, et al. Understanding the linkage between urban growth and land surface temperature—A case study of Bangalore City, India. Remote Sens. 2022;14(17):4241. doi: 10.3390/rs14174241

[68] Yin CL, Meng F, Yu QR. Calculation of land surface emissivity and retrieval of land surface temperature based on a spectral mixing model. Infrared Phys Technol. 2020;108:103333. doi: 10.1016/j.infrared.2020.103333

[69] Sekertekin A, Bonafoni S. Land surface temperature retrieval from Landsat 5, 7, and 8 over rural areas: assessment of different retrieval algorithms and emissivity models and toolbox implementation. Remote Sens. 2020;12(2):294. doi: 10.3390/rs12020294

[70] Li Z, Wu H, Duan S, Zhao W, Ren H, Liu X, et al. Satellite remote sensing of global land surface temperature: definition, methods, products, and applications. Rev Geophys. 2023;61(1). doi: 10.1029/2022rg000777

[71] Ferguglia O, Palazzi E, Arnone E. Elevation dependent change in ERA5 precipitation and its extremes. Clim Dyn. 2024;62(8):8137–53. doi: 10.1007/s00382-024-07328-6

[72] Johannsen F, Ermida S, Martins JPA, Trigo IF, Nogueira M, Dutra E. Cold Bias of ERA5 summertime daily maximum land surface temperature over Iberian Peninsula. Remote Sens. 2019;11(21):2570. doi: 10.3390/rs11212570

[73] Ganguli P, Merz B. Increasing probability of extreme rainfall preconditioned by humid heatwaves in global coastal megacities. npj Clim Atmos Sci. 2025;8(1). doi: 10.1038/s41612-025-01023-x

[74] Roffe SJ, van der Walt AJ. Representation and evaluation of southern Africa’s seasonal mean and extreme temperatures in the ERA5-based reanalysis products. Atmospheric Res. 2023;284:106591. doi: 10.1016/j.atmosres.2022.106591

